# Supplementary material for: MightyU – A portable sensor-based video game application for exercise training of children and adolescents with cerebral palsy
Source: PLoS One. 2026 Feb 4;21(2):e0339704. doi: 10.1371/journal.pone.0339704 (PMC12872008; doi:10.1371/journal.pone.0339704)
Supplement: S1 Table — (PDF) [file pone.0339704.s001.pdf]

MightyU: User observation questionnaire

Children's version

Game Experience Questionnaire - GEQ (modified and shortened)

		not at all	*slightly*	*moderately*	*fairly*	*extremely*
1	I felt content	*O*	*O*	*O*	*O*	*O*
2	I felt skilful	*O*	*O*	*O*	*O*	*O*
3	I was interested in the game's story	*O*	*O*	*O*	*O*	*O*
4	I thought it was fun	O	O	O	O	O
(5)	*I was fully occupied with the game*	*O*	*O*	*O*	*O*	*O*
(6)	*I felt happy*	*O*	*O*	*O*	*O*	*O*
7	It gave me a bad mood	*O*	*O*	*O*	*O*	*O*
(8)	*I thought about other things*	*O*	*O*	*O*	*O*	*O*
9	I found it tiresome	*O*	*O*	*O*	*O*	*O*
10	I felt competent	*O*	*O*	*O*	*O*	*O*
11	I thought it was hard	*O*	*O*	*O*	*O*	*O*
12	It was aesthetically pleasing	*O*	*O*	*O*	*O*	*O*
13	I forgot everything around me	*O*	*O*	*O*	*O*	*O*
(14)	*I felt good*	*O*	*O*	*O*	*O*	*O*
(15)	*I was good at it*	*O*	*O*	*O*	*O*	*O*
16	I felt bored	*O*	*O*	*O*	*O*	*O*
(17)	*I felt successful*	*O*	*O*	*O*	*O*	*O*
(18)	*I felt imaginative*	*O*	*O*	*O*	*O*	*O*
(19)	*I felt that I could explore things*	*O*	*O*	*O*	*O*	*O*
(20)	*I enjoyed it*	*O*	*O*	*O*	*O*	*O*
(21)	*I was fast at reaching the game's targets*	*O*	*O*	*O*	*O*	*O*
(22)	*I felt annoyed*	*O*	*O*	*O*	*O*	*O*
(13)	*I felt pressured*	*O*	*O*	*O*	*O*	*O*
(24)	*I felt irritable*	*O*	*O*	*O*	*O*	*O*
(25)	*I lost track of time*	*O*	*O*	*O*	*O*	*O*
26	I felt challenged	*O*	*O*	*O*	*O*	*O*

(27)	*I found it impressive*	*O*	*O*	*O*	*O*	*O*
28	I was deeply concentrated in the game	*O*	*O*	*O*	*O*	*O*
(29)	*I felt frustrated*	*O*	*O*	*O*	*O*	*O*
(30)	*It felt like a rich experience*	*O*	*O*	*O*	*O*	*O*
(31)	*I lost connection with the outside world*	*O*	*O*	*O*	*O*	*O*
(32)	*I felt time pressure*	*O*	*O*	*O*	*O*	*O*
(33)	*I had to put a lot of effort into it*	*O*	*O*	*O*	*O*	*O*
*1	I felt dizzy or nauseous	*O*	*O*	*O*	*O*	*O*
*2	I liked playing together	*O*	*O*	*O*	*O*	*O*
*3	I often didn't know what to do	*O*	*O*	*O*	*O*	*O*
*4	I had muscle/joint pain while playing	*O*	*O*	*O*	*O*	*O*

Original questionnaire of GEQ (questions 1-33). The italic typed questions were excluded. Questions marked with an * have been added and are not part of the GEQ. Source: IJsselsteijn, W. A., de Kort, Y. A. W., & Poels, K. (2013). The Game Experience Questionnaire. Technische Universiteit Eindhoven.

System Usability Scale - SUS (questions 1-3 from 10)

1. I think that I would like to use this system frequently.

**Strongly Disagree 1**	**2**	**3**	**4**	**Strongly Agree 5**
○	○	○	○	○

2. Ich found the system unnecessarily complex.

**Strongly Disagree 1**	**2**	**3**	**4**	**Strongly Agree 5**
○	○	○	○	○

3. Ich thought the system was easy to use.

**Strongly Disagree 1**	**2**	**3**	**4**	**Strongly Agree 5**
○	○	○	○	○

Original questionnaire of SEQ (questions 1-3). Source: Brooke, J. (1996). SUS: A ‘quick and dirty’ usability scale. In Jordan, P.; Thomas, B.; Weerdmeester, B. & McClelland, I. L. (ed.) Usability Evaluation in Industry. S. 189-194, Taylor & Francis.
