# Supplementary material for: MightyU – A portable sensor-based video game application for exercise training of children and adolescents with cerebral palsy
Source: PLoS One. 2026 Feb 4;21(2):e0339704. doi: 10.1371/journal.pone.0339704 (PMC12872008; doi:10.1371/journal.pone.0339704)
Supplement: S2 Table — (PDF) [file pone.0339704.s002.pdf]

MightyU: User observation questionnaire

Parent's version

(modified for parents)

Game Experience Questionnaire - GEQ (modified and shortened)

		not at all	*slightly*	*moderately*	*fairly*	*extremely*
1	My child felt content	*O*	*O*	*O*	*O*	*O*
2	My child felt skilful	*O*	*O*	*O*	*O*	*O*
3	My child was interested in the game's story	*O*	*O*	*O*	*O*	*O*
4	My child had fun	O	O	O	O	O
7	It gave my child a bad mood	*O*	*O*	*O*	*O*	*O*
9	My child found it tiresome	*O*	*O*	*O*	*O*	*O*
10	My child felt competent	*O*	*O*	*O*	*O*	*O*
11	My child thought it was hard	*O*	*O*	*O*	*O*	*O*
12	It was aesthetically pleasing	*O*	*O*	*O*	*O*	*O*
13	My child forgot everything around	*O*	*O*	*O*	*O*	*O*
16	My child felt bored	*O*	*O*	*O*	*O*	*O*
26	My child felt challenged	*O*	*O*	*O*	*O*	*O*
28	My child was deeply concentrated in the game	*O*	*O*	*O*	*O*	*O*
*1	My child felt dizzy or nauseous	*O*	*O*	*O*	*O*	*O*
*2	My child liked playing together	*O*	*O*	*O*	*O*	*O*
*3	My child often didn't know what to do	*O*	*O*	*O*	*O*	*O*
*4	My child had muscle/joint pain while playing	*O*	*O*	*O*	*O*	*O*

Modified GEQ questionnaire with additional questions. Original source: IJsselsteijn, W. A., de Kort, Y. A. W., & Poels, K. (2013). The Game Experience Questionnaire. Eindhoven University of Technology.

System Usability Scale - SUS (questions 1-3 from 10)

1. I think that I would like to use this system frequently.

Strongly Disagree 1	2	3	4	Strongly Agree 5
○	○	○	○	○

2. Ich found the system unnecessarily complex.

Strongly Disagree 1	2	3	4	Strongly Agree 5
○	○	○	○	○

3. Ich thought the system was easy to use.

Strongly Disagree 1	2	3	4	Strongly Agree 5
○	○	○	○	○

Shortened SEQ questionnaire, self-translated (questions 1-3). Original source: Brooke, J. (1996). SUS: A 'quick and dirty' usability scale. In Jordan, P.; Thomas, B.; Weerdmeester, B. & McClelland, I. L. (ed.) Usability Evaluation in Industry. pp. 189-194, Taylor & Francis.

Additional questions:

Have you observed any short-term therapeutic effects in your child?

Please list below:

___

___

___

___

Have you observed any side effects in your child?

Please list below:

___

___

___

___
