# Supplementary material for: MightyU – A portable sensor-based video game application for exercise training of children and adolescents with cerebral palsy
Source: PLoS One. 2026 Feb 4;21(2):e0339704. doi: 10.1371/journal.pone.0339704 (PMC12872008; doi:10.1371/journal.pone.0339704)
Supplement: Attachment — Submitted filename: Reply_Reviewer.pdf [file pone.0339704.s005.pdf]

Katholisches Klinikum Bochum

St. Josef-Hospital
UK RUB UNIVERSITÄTSKLINIKUM DER RUHR-UNIVERSITÄT BOCHUM

Universitätsklinik für
Kinder- und Jugendmedizin
Direktor
Univ.-Prof. Dr. med. Thomas Lücke

Sandra Kühn (Sekretariat)
Telefon 0234/509-2611
Telefax 0234/509-2612
sandra.kuehn@klinikum-bochum.de
www.kinderklinik-bochum.de

Mighty U – A portable sensor-based video game application for exercise training of children and adolescents with cerebral palsy

Authors: Lynn Eitner, Lennart Lücke, Elnaz Farshadfar, Christian Grüneberg, Christoph Maier, Almut Weitkämper, Bettina Menzen, Anja Burmann, Roman von Gehlen, Peter Konrad, Thomas Immich, Britta Karn, Patrick Filipowicz, Maximilian Pilk, Thomas Lücke

Reply to reviewers

Reviewer #1:

Introduction

Would it be worth adding some comment on this being a form of biofeedback (and what it known about biodfeedback)? Around line 63 it might be relevant to mention biofeedback here.

Author's reply:

Thank you for the idea of making the very useful connection to biofeedback. We will now explain this method, provide an insight into how biofeedback is used in the neurorehabilitation of children, and establish a connection to our training device. (page 5, lines 65-71)

Methods

How did you decide on which muscles to put the sensors on for each person (i.e. how did the physiotherapists decide on this)? Where the sensors placed across all of the muscle sites lists or were they targeted to the child? How did you ensure that the participants places the sensors on the correct locations at home? some of these muscles can be quite small, even more so on kids with CP- how do you avoid cross talk?

It is hard to work out how many participants had one or more sensors on them, or what combination of muscles they had tested at time, could this be explained (maybe table 2 could have more explanation?)

Author's reply:

This feedback is also very helpful. We have now added a subsection entitled "Muscle selection and sEMG sensor placement" to the methods section to describe these points in more detail. (page 10)

Results

Do you have data on how long they used the system each time (minutes?), then also some sort of indication on the number of repetitions they might have made in a particular movement/contraction? This would greatly strengthen this work.

Author's reply:

We added how many days the children used the system for training at home. Unfortunately, exact minute details cannot currently be extracted.

Is there any option to also include some of the EMG or kinematic data within this manuscript? and how

did it relate to the muscles that were needing to be targeted (e.g. in some cases it is not the muscle that we are aiming to recruit that is used to complete a task).
Author's reply:
Three reviewers suggest including sEMG data in this manuscript (see below). Our original plan was to focus solely on the feasibility and usability of the new video game application in this publication. The motion analyses, which, in addition to sEMG data, also include kinematic data from marker-based videogrammetry (2D) and a measurement using an inertial sensor system (IMU) (3D), were intended to be the subject of a subsequent publication. We have now decided to include the topic of sEMG in this publication as an example (see figure 5).

Discussion
It feels like this is getting a little away from the application (and principles) of rehabilitation. Yes - specific task training is needed, and it generally applies to functional tasks based on the child's therapy goals, that also need to active (not passive) – which is where the emg biofeedback approach is a positive step. "Engineer build training applications" could be misinterpreted here as being the solution.
Author's reply:
Thank you for this important comment. We certainly don't want the video game to be misunderstood as a replacement for all conventional and proven therapy options. It is simply intended to be a child-friendly and motivating addition to everyday therapy. We have clarified the relevant passages.

Can the game within the system be modified to suit children with higher GMFCS levels? These are the children who could likely benefit the most.
Author's reply:
This option is desirable for further development of the system. For the initial test runs, we needed a system that is as broadly applicable as possible, so that the largest possible patient group can try out the application and adequately evaluate it.

It might be important to comment that future work would be needed to evaluate if there are any functional changes with this tool.
Author's reply:
Thank you for this idea, we added this hint to the conclusions.

Reviewer #3:
ABSTRACT
Lines 8-9: Could you clarify what you mean by "kinematic electromyography"? Are you referring exclusively to surface EMG?
Author's reply:
We agree with the reviewer that the term is misleading. Therefore, we have removed it from the text passage and defined it as follows: "A surface electromyography sensor detects muscle activation, which is translated into in-game actions. In this way, targeted muscle activity is used to collect coins during gameplay." (page 2, lines 8ff)

METHODS
Provide a more detailed description of the movements and which muscles were targeted. Specify the anatomical points where sensors were placed. Specify the type of muscles or muscle groups targeted. Line 132: Specify which muscles were involved and explain how you determined if the maximum contraction was performed by the child.
Author's reply:
Thank you for feedback. We added in the section methods additional information concerning selected muscles and positions sensors applied and the target movement in table 2.

Explain the calibration procedure or provide a reference for it.
Author's reply:
We appreciate your input and enhance the explanation in the method section with additional definition on sensor calibration procedure for the individual patients. (page 10, lines 169ff)

Clarify what criteria were used to add an obstacle and how many more obstacles were quantified.
Author's reply:
Thanks for your raising this topic. We added additional information on the same standardized level all included patients played during the study visits. This level did not have any obstacles included. (page 11, lines 184ff)

Specify which types of variables were analyzed and clarify which variables were measured in the study.
Author's reply:
Thanks for your feedback and input. In this first manuscript we are only reviewing the feasibility and usability of the new training assessment. As measuring tool a survey has been used among the children and parents. We now explain the statistical procedures in more detail in the results section. (pages 15ff)

DISCUSSION

Lines 356-357: "Allowed the intensity of muscle contraction to be quantified"—How was this quantified? Please clarify the methodology.
Author's reply:
Thanks for your comment. The intensity of contraction can be monitored during the gameplay by reviewing the movement of the character in the game. Compare explanations on biofeedback in the introduction. (page 5, lines 62ff and page 9, lines 148ff)

Explain the limitations of the study and the potential for future developments more clearly.
Author's reply:
We appreciate your feedback. Limitations, improvement need and further development potentials in different areas have been stated in the discussion section.

FIGURES

Figure 2: Add the statistical analysis performed to the figure.
Author's reply:
Thanks for your feedback, we have added percentages of the positive and negative answers to get an overview about the frequency. Explanations to the figures are indicated under Feasibility. (see figure 3)

GENERAL CONSIDERATIONS

Revise the "Methods" section to use a more scientific tone. Some points could benefit from rephrasing to improve clarity.
Author's reply:
Several sections have been updated accordingly.

Provide the results with the respective statistical analyses.
Author's reply:

Statistical analyses have been added.

Clarify the type of variables used to control the game—only EMG amplitude?
Author's reply:
This is correct, only EMG sensor has been used.

Add a photo of the experimental set up.
Author's reply:
We added a photo of the EEG-sensor located on a child's forearm. (see Fig.1)

Reviewer #4:

1.The authors failed to mention any information regarding how their device or game improved the scientific or theoretical knowledge of treatment or therapeutic/rehabilitatory observations of their patient under study.
Author's reply:
We can agree with this objection. The patients only tested the training tool at home for one week, and the initial aim of this intervention was to test its usability and feasibility. The next step is to assess its therapeutic benefit. However, we are, of course, aware that the intervention phase will need to last significantly longer. It is part of a further study.

2.The game includes the sensors of EMG, that could have been their main focused since it was the bridge between virtual game development and scientific advancement in neuro-therapeutic field. There was no data provided in the current form of this article in this regard. In the discussion section authors promised to provide that in their future publications. I think it would be good to combine their present and future goals together to establish a story with good scientific merit.
Author's reply:
Same topic raised by other reviewer 1. Compare above.

Concerns related to the current form of the article:
1. In the introduction, authors mentioned modification in the MightyU project (Page 5 line 69): Is the program upgradable? Meaning, is it possible to modify or adjust the difficulty level of the game in accordance with patients' impairments and/or improvement? More justification would be appreciated for the readers.
Author's reply:
Same topic raised by other reviewer. Compare above

2. Method section (page 6 line 80) suggests that the project has been approved by the ethics committee in 2020. The obvious question comes, why has there not been any follow up study conducted? --?! :D
Author's reply:
Thanks for your feedback. There has been a lot of interest in a follow up study from various parties. Planning activities are ongoing.

3. The game development section (Page 7 line 113) described the game. What is unique about the logic/algorithm of the game which makes this development unique and tailored to the patient as compared to their competitors' product? Little more description would be helpful for the reader to understand the rational.
Author's reply:

Thank you for this comment. We added this information in the introduction. (page 5, lines 61ff)

4. The section also talks about the use of EMG in the playing process and gaming score. The patient needs to contract or relax their muscles to keep the virtual player floating on the screen during the game. Can authors comment on whether the recording from EMG data pattern and the gaming score comparable? It would be helpful to directly relate their gamification process with therapeutic progress.

Author's reply:

We have included an example to illustrate the EMG analysis, which several reviewers requested. See Figure 6 and comments page 20, lines 341ff.

5. Measurement procedure (Page 10 line 190): Was the EMG data collected during the training, practice or trail sessions being used in medical research in any form? Was the data evaluated by the patient's therapist? Detailed description of whether or how that data has been processed will give the article a justification for developing muscle targeted gaming device for CP patients. (line 205)

Author's reply:

See question above. The next step is to assess the advices therapeutic benefit.

6.Figure 3 required more explanation why the GEQ rating of healthy parents and children with CP are almost identical in terms of competence and challenge parameters.

Author's reply:

Thank you for your feedback. Further explanations have been added to the manuscript. Parents' responses are based on observations while their children were using the game application. (page 17, line 293)

7.Figure 4: The comparative line graphs show almost 70% cases where the GMFC score improved from 1st to 2nd assessment. Based on the gaming logic explained in this article, neurologically that graph implies strengthening of muscle or improvement on controlling muscle contraction/relaxation. These are very important parameters for CP therapeutic aspect. The question raises why this result has not been correlated with EMG data collected during the assessments?

Author's reply:

Three reviewers suggest including sEMG data in this manuscript (see above). Our original plan was to focus solely on the feasibility and usability of the new video game application in this publication. The motion analyses, which, in addition to sEMG data, also include kinematic data from marker-based videogrammetry (2D) and a measurement using an inertial sensor system (IMU) (3D), were intended to be the subject of a subsequent publication. We have now decided to include the topic of sEMG in this publication as an example (see figure 6).

Reviewer #5:

Thank you for this submission. This is an interesting study and the MightyU seems like a valuable addition to a home exercise program.

**While the study appears to be sound, the language is sometimes unclear, making it difficult to follow. I advise the authors work with a writing coach or copyeditor to improve the flow and readability of the text.*

Author's reply:

We appreciate your feedback. Several passages of the manuscript have been updated to simplify and be more sound on the argumentation structure.

**I would not call the MightyU a VR program. It is a game, but not virtual reality based on your description.*
Author's reply:
Thanks for the very valuable comment. It has been corrected.

**It would be interesting to see if the participants demonstrated increased contraction strength as a result of their use of the device. Is this information available or will this be a future study?*
Author's reply:
Children only used the Game for a rather short duration of one week, therefore we did not intend to review improvement contraction strength. For a future study this investigation will be in scope.

**How often should this be re-calibrated during their use of the product? If I understand correctly, the device is calibrated to the participant once when treatment is initiated and then was not re-calibrated during the one-week trial.*
Author's reply:
Thanks for your feedback. Additional information on the calibration procedure has been added to clarify this point. (page 10, lines 170ff)

Reviewer #6: Overall summary
1) The manuscript isn't very sound because it doesn't fully decribe nor explain the experimental setup. Moreover, it doesn't include any clinical outcome measure nor controls of any sort. No hypotheses are made and therefore data can neither support nor reject the hypotheses.
Author's reply:
We thank you for your review of the manuscript. The methodology was significantly expanded and clarified during the revision. The initial evaluation focused on assessing the feasibility and usability of the novel game application. For this purpose, questionnaires were conducted with children and their observing parents. Therefore, the assessment of the actual target group—children with cerebral palsy—was of interest, so no control group was planned in this first step.

2) Statistical analysis is virtually absent, since data are only descriptive. Some analysis has been carried out, but no conclusions can be drawn.
Author's reply:
On this point, we do not entirely agree with the reviewer. Of course, with a sample size of 21 children (17 fully completed questionnaires), a comprehensive statistical analysis is not possible, but a descriptive evaluation. Nevertheless, this is a common method for generating results that also allow first conclusions and summaries.

3) I couldn't find the full clinical description of cases, nor the association between clinical pictures (type, distribution of motor disorders) and experimental conditions.
What CP type (topography and type of motor disorder) was matched with what muscles? Clinical characterisation is very poor.
Author's reply:
We thank you for rising this issue. Clinical characterizations of the patient group have been added (Table 1), and the methods now explain in more detail the criteria used to select the target movements and muscle groups.

4) The manuscript is written in standard English, but it hasn't been revised.
Author's reply:
Thank you for your feedback. Many passages has been revised.

Methods

Page 7, line 117. What is special about the sensors?

Author's reply:

We added information about the sensors under the section "the game". (page 9, lines 138ff)

Page 7, line 120. Setup specifications are not clear enough. Please add technical data such as latency and measurement errors. Please specify how many sensors were used and in which configuration. Cross-talk issues? Latency issues? Was the bluetooth connection for each sensor or were sensors cabled to a single transmitter?

Author's reply:

Thank you for this comment. Methods have been updated accordingly the sensors and we added a photo of the EEG-sensor located on a child's forearm.

By the way, does each child use always the same muscle(s) for gearing the game? Does each child use one or more muscles at a time? Is game duration always the same or does it change according to levels? Procedure requires clarification.

Author's reply:

Thank you for this inquiry. We have expanded the methodology at this point to include some explanations. Due to the one-week training period and for better comparability, we selected one target movement for each child that was not varied. We have also made improvements to the descriptions of the training levels.

Page 7, line 122. Define 'muscle tension': which signal measure was used and how? Error?

Author's reply:

We have clarified this passage as follows: "Depending on the extend of muscle contraction (amplitude of the EMG signal), the character´s position can be influenced." (page 9, lines 148ff)

Page 7, line 123. Explain the calibration procedure.

Author's reply:

Thank you for this idea, we enhanced the explanation in the method section with additional definition on sensor calibration procedure for the individual patients.

Page 9, line 170. How did you normalise the measure 'number of coins' across different levels, game durations (if variable), and muscles tested? Why didn't you investigate any clinical outcome measure, such as muscle strength, endurance, gross-motor function, attention, QoL?

Author's reply:

More explanations on the standardized training level added from line 199 onwards. Deeper clinical studies on physical changes have not being carried, as the study was primary focusing on the feasibility and usability of the tool. In addition to this, with the one week training period not significant physical changes would be expected.

Discussion

I expected to gain more insight into the pros and contras of this particular methodology in comparison with other 1) types of exergame, 2) types of sensorisation, 3) training methods.

Author's reply:

Thank you for the feedback. Please review line 415 onwards we discussed the advantages and benefits of the sensors.
