# Supplementary material for: MightyU – A portable sensor-based video game application for exercise training of children and adolescents with cerebral palsy
Source: PLoS One. 2026 Feb 4;21(2):e0339704. doi: 10.1371/journal.pone.0339704 (PMC12872008; doi:10.1371/journal.pone.0339704)
Supplement: Attachment — Submitted filename: Letter to the Editor for PLOS ONE_PONE-D-24-54773.docx [file pone.0339704.s003.docx]

Dear Editor,	February 21^st^, 2025

Thank you for the opportunity to serve PLOS One journal as a reviewer.

The manuscript under consideration entitled ‘Mighty U – A portable sensor-based video game application for exercise training of children and adolescents with cerebral palsy’ (ID: PONE-D-24-54773) is a very interesting approach in neuro- rehabilitory therapeutic field. Lynn Eitner and the group took the most trending virtual reality approach to develop a gamming concept as a therapeutic or rehabilitatory tool for cerebral palsy patients. To edge their virtual gaming competitive market, the authors combined sensor-based surface EMG technology as the human operating control for the video game, which can detect and record small muscle contractions over time. This gave the authors to tailor the game specifically for patient needs or disability, targeting muscle of interest which made the tool versatile. I really appreciate their thought process, and the authors conceptually contributed towards the merit of personalized therapeutic research. But I think Lynn Eitner *et al* under delivered on their aims and goals in terms of their results and observations. I found lots of missed opportunities to upgrade the article in scientific merit to fit into PLOS One journal.

Thus, **I would not recommend** the current version of the manuscript suitable for publication in ‘PLOS One’.

Here I would like to address a few of the important areas of my concern to support my discission and highlighting the scope of improvement for the authors.

**Overall concerns:**

1. The authors designed their manuscript around the virtual reality game, its development, tailoring to the patient needs, surveys, etc, but the aim of the article suggests ‘… exercise training of the children and adolescents with cerebral palsy’. They failed to mention any information regarding how their device or game improved the scientific or theoretical knowledge of treatment or therapeutic/rehabilitatory observations of their patient under study.
2. The game includes the sensors of EMG, that could have been their main focused since it was the bridge between virtual game development and scientific advancement in neuro-therapeutic field. There was no data provided in the current form of this article in this regard. In the discussion section authors promised to provide that in their future publications. I think it would be good to combine their present and future goals together to establish a story with good scientific merit.

**Concerns related to the current form of the article:**

1. In the introduction, authors mentioned modification in the MightyU project (Page 5 line 69): Is the program upgradable? Meaning, is it possible to modify or adjust the difficulty level of the game in accordance with patients’ impairments and/or improvement? More justification would be appreciated for the readers.
2. Method section (page 6 line 80) suggests that the project has been approved by the ethics committee in 2020. The obvious question comes, why has there not been any follow up study conducted?
3. The game development section (Page 7 line 113) described the game. What is unique about the logic/algorithm of the game which makes this development unique and tailored to the patient as compared to their competitors’ product? Little more description would be helpful for the reader to understand the rational.
4. The section also talks about the use of EMG in the playing process and gaming score. The patient needs to contract or relax their muscles to keep the virtual player floating on the screen during the game. Can authors comment on whether the recording from EMG data pattern and the gaming score comparable? It would be helpful to directly relate their gamification process with therapeutic progress.
5. Measurement procedure (Page 10 line 190): Was the EMG data collected during the training, practice or trail sessions being used in medical research in any form? Was the data evaluated by the patient’s therapist? Detailed description of whether or how that data has been processed will give the article a justification for developing muscle targeted gamming device for CP patients.
6. Figure 3 required more explanation why the GEQ rating of healthy parents and children with CP are almost identical in terms of competence and challenge parameters.
7. Figure 4: The comparative line graphs show almost 70% cases where the GMFC score improved from 1^st^ to 2^nd^ assessment. Based on the gaming logic explained in this article, neurologically that graph implies strengthening of muscle or improvement on controlling muscle contraction/relaxation. These are very important parameters for CP therapeutic aspect. The question raises why this result has not been correlated with EMG data collected during the assessments?

Overall, I appreciate the effort the authors put forward. The study in current form is premature and there is a huge opportunity to improve this study in scientific merit.

Thank you for the opportunity and looking forward to serving the journal again.

Sincerely,

Srikanya

Srikanya Kundu, PhD

Neuroscientist, NCATS/NIH

3D-Bioprinting Group, Division of Pre-Clinical Innovation

9800 Medical Center Drive

Rockville, MD 20850
