# Supplementary material for: MightyU – A portable sensor-based video game application for exercise training of children and adolescents with cerebral palsy
Source: PLoS One. 2026 Feb 4;21(2):e0339704. doi: 10.1371/journal.pone.0339704 (PMC12872008; doi:10.1371/journal.pone.0339704)
Supplement: Attachment — Submitted filename: Reply_Reviewer_20251119.docx [file pone.0339704.s006.docx]

| Universitätsklinik für  Kinder- und Jugendmedizin  Direktor  Univ.-Prof. Dr. med. Thomas Lücke  _______________________________  Sandra Kühn (Sekretariat)  Telefon 0234/509-2611  Telefax 0234/509-2612  sandra.kuehn@klinikum-bochum.de  www.kinderklinik-bochum.de |  |
| --- | --- |
|  |  |

**Mighty U – A portable sensor-based video game application for exercise training of children and adolescents with cerebral palsy**

Authors: Lynn Eitner, Lennart Lücke, Elnaz Farshadfar, Christian Grüneberg, Christoph Maier, Almut Weitkämper, Bettina Menzen, Anja Burmann, Roman von Gehlen, Peter Konrad, Thomas Immich, Britta Karn, Patrick Filipowicz, Maximilian Pilk, Thomas Lücke

**Reply to reviewers**

Thank you very much for your support, your ideas that have greatly enriched the manuscript, and the time you have taken to edit it.

**Reviewer #3:**

*Abstract
break long sentences, clarify the effect size (68% improvement).
Include the number of participants in the Methods section of the abstract.*

**Author’s reply:**

Thank you very much for these comments. We have revised the text passages and shortened the sentences.

*Terminology and consistency
Muscles:
quadrizeps femoris → quadriceps femoris
digitorum profundus palmarflexion → flexor digitorum profundus – wrist/finger flexion
peroneus longus abduction → peroneus longus – foot eversion
Correlation coefficient: replace “onkoeffic ient”.
Standardize abbreviations: sEMG, EMG.*
**Author’s reply:**

Thank you very much for the suggestions. We have adopted them all.

*Methods
Break long sentences in the EMG sensor and gameplay descriptions.
Clarify access to data on the web portal.
line 223: remove) at the end of the sentence*

**Author’s reply:**

Thank you very much for these comments. We shortened the sentences (page 9, lines 153ff) and added a description of data assessment on the web portal (page 10, lines 198ff).

*Use bullet points for technical parameters (A/D conversion, filtering, RMS).*

**Author’s reply:**

*This is a good suggestion. We tried bullet points but found that reducing the descriptions greatly limited comprehensibility. For this reason, we have retained complete sentences.*

*Clearly describe the GEQ and SUS scales.*

**Author’s reply:**

We have added a reference to the material in the supplements. This contains all the questions from both questionnaires.

Results
*Correct muscle names and movements in Table 2.*
**Author’s reply:**

Thank you very much. We have implemented this suggestion and completely revised Table 2.

*Standardize data presentation across text, tables, and figures (percentages vs. medians).*
**Author’s reply:**

We have checked all texts, tables, and illustrations for discrepancies and have not found any inconsistencies.

*Check discrepancies between the number of participants reported in text and figures.*

**Author’s reply:**

All evaluations refer to the 16 participants who took part in both visits. Therefore, all tables and figures include 16 children. Nevertheless, it was relevant for the results of the study to also mention the others, those who did not want to participate and those who only took part in the first visit. We describe this in the text on page 14, lines 257 ff.

Discussion
*Some sections are redundant (e.g., paragraphs 356–398).
Avoid repetition of previously reported results.*

**Author’s reply:**

We appreciate this comment, but we believe that it is usual to summarize the results again at the beginning of the discussion for the sake of readability and to provide a quick overview. We think that it is helpful for the flow of reading. For this reason, we have decided not to delete the passages.

*line 389 avoid to use patients. Children should be better.
Explain the limitations of the study and the potential for future developments more clearly.*
**Author’s reply:**

We have incorporated these comments and added some explanatory sentences to the discussion (page 24, lines 473 ff).

Conclusions
*Simplify long sentences.
Summarize in 2–3 concise sentences: safety, usability, motivation, potential for home-based training.*

**Author’s reply:**

We shortened the conclusion a bit and removed long sentences.

Tables and figures
*Table 2: correct muscle names and movements.
Figures 1–6: check readability and legends; standardize numbering.
GEQ/SUS: standardize style and units.
Table 3: replace a comma with . 0,32 -> 0.32
replace correlationkoefficent with "correlation coefficient"
Figure 5: add "S" to "GMFC"*
**Author’s reply:**

This feedback was very helpful. We have implemented all points in the tables and figures.

*Standardize verb tense: past for methods, present for results and interpretation. Check punctuation, spacing, and terminological consistency.*

**Author’s reply:**

Thank you very much for this tip. We have made the necessary corrections.

TABLE 2
*I would correct the table as follows.
Biceps brachii Flexion of the forearm
Brachioradialis Flexion of the forearm / assists supination
Flexor digitorum profundus Flexion of the fingers
Extensor carpi radialis longus Extension of the wrist
Extensor digitorum Extension of the fingers
Tibialis anterior Dorsiflexion of the foot
Quadriceps femoris Extension of the knee joint
Peroneus longus Eversion of the foot*

**Author’s reply:**

We appreciate this excellent suggestion and have incorporated everything into the table exactly as you suggested.

**Reviewer #4:**

All comments were address to the best of their ability and available data. The current manuscript has been considered as a first part of their ongoing reserach. Looking forward to their upcoming publication.

**Author’s reply:**

We would like to thank you for your support.

**Reviewer #5:**

Thank you for the opportunity to review this resubmission. A few minor comments. there is a typo in Table 3 Correlation Coefficient.
Figure 5, left axis of the figure is hard to read, recommend labeling every 5 coins.
This is a much better submission than the original. Thank you for addressing our concerns.

**Author’s reply:**

Thank you very much for pointing this out. We have corrected it accordingly.
